# Engaging veterans in the development of mobile health interventions to support medication treatment for opioid use disorder

**DOI:** 10.3389/fdgth.2026.1805086

**Published:** 2026-09-04

**Authors:** Noah R. Wolkowicz, Christie W. Musket, Erin D. Reilly, Eric Kuhn, Mehmet Sofuoglu, R. Ross MacLean, Galina A. Portnoy

**Affiliations:** 1 Research Service Line, VA Connecticut Healthcare System, West Haven, CT, United States; 2 Department of Psychiatry, Yale School of Medicine, New Haven, CT, United States; 3 Research Service Line, VISN1 Mental Illness Research, Education, and Clinical Center (MIRECC), West Haven, CT, United States; 4 Columbia University Medical Center, Morningside Heights, NY, United States; 5 Mental Health Service Line, VA Bedford Healthcare System, Bedford, MA, United States; 6 University of Massachusetts Chan School of Medicine, Wrocester, MA, United States; 7 National Center for PTSD, Dissemination and Training Division, VA Palo Alto Health Care System, Palo Alto, CA, United States; 8 Department of Psychiatry and Behavioral Sciences, Stanford University School of Medicine, Stanford, CA, United States

**Keywords:** addiction, digital health, m-healfh, mobile health (mHealth), opioid, qualitative, rapid qualitative analysis, veteran

## Abstract

**Introduction:**

Opioid Use Disorder (OUD) is an ongoing and pervasive public health problem. Despite the availability of efficacious Medication treatments for OUD (MOUD), many patients require additional support to address OUD's wide-ranging consequences. Mobile Health Interventions (MHIs), or smartphone-based software applications (“apps”), could serve as a scalable and flexible medium to increase adjunctive care access within MOUD.

**Method:**

As an initial step in potential MHI creation, we conducted semi-structured interviews with U.S. military veterans receiving MOUD (*N* = 21) via rapid qualitative methods. Our team developed a start-list of interview questions. Interview transcripts were independently coded by interviewers, who then convened to discuss and refine code definitions, develop summary templates, organize codes into hierarchical structures, and refine domain categories through consensus.

**Results:**

Twenty-one veterans participated [M[SD]Age = 59.95[10.50]; 90.48% male sex-assigned-at-birth; 80.95% White; 85.71% Non-Hispanic/Latinx; 52.38% on buprenorphine, 47.62% on methadone at time of interview]. Veteran attitudes were typically either neutral- positively valenced and characterized by willingness (Open Prudency) or neutral-negatively valenced and characterized by concern and contingencies for use, such as assurance for continued access to in-person care (Hesitancy). A small but notable proportion of veterans expressed strongly favorable views towards MHIs, often grounded in their own technological fluency (Enthusiasm). Outright Opposition to MHIs was rare. Desired MHI functions were variable and included their use for enhancing care accessibility and convenience (e.g., Resource Centralization, Patient-Provider Communication), as well as supporting autonomy, motivation and individualization (e.g., Self-Monitoring, Positive Reinforcement, Adjunctive Intervention).

**Conclusions:**

Results from this qualitative investigation of U.S. military veterans provide foundational data to guide future development of MHIs for supporting MOUD engagement. Findings suggest that veterans receiving MOUD are generally open towards and interested in using MHIs as part of their care regimens, which were broadly described as potential means to enhance treatment accessibility, convenience, and individualization.

## Introduction

Opioid Use Disorder (OUD) is a pervasive and ongoing public health crisis, responsible for the deaths of over 806,000 (1) people between 1999 and 2023 and with annual healthcare costs exceeding $1 trillion (2, 3). OUD is associated with severe impairment across many facets of daily life, such as greater rates of medical and psychiatric comorbidities (4), reduced health-related quality of life (5, 6), homelessness (7), and unemployment (8). Such harms contribute to staggering economic burdens on the U.S. healthcare system, which were in excess of nearly $1.5 trillion in 2020 alone (3). OUD and its related harms are no less problematic among the U.S. military veteran community (2, 9–12), who also tend to show higher rates of co-occurring problems like pain (13), and PTSD (14), and other forms of substance misuse (15) than their civilian counterparts. Despite significant nationwide efforts to address OUD (16–18) and recent decreases in overdose trends (19), opioid overdose deaths remain shockingly high with ∼80,000 recorded in 2023 (19). Advances in OUD treatment are needed to further address the ongoing consequences of OUD.

Efficacious Medication treatments for Opioid Use Disorder (MOUD; e.g., methadone, buprenorphine) are available and MOUD can reduce overdose mortality rates by up to ∼70% (buprenorphine) and 79% (methadone) (20–23). MOUD is recommended as the first line of care across numerous OUD clinical practice guidelines, including within the Veterans Health Administration (VHA) (21, 24). Nonetheless, engagement with MOUD regimens can be difficult (25–28). MOUD is often implemented via long-term regimens [consistent with OUD's conceptualization as a chronic illness (29)], with recommended durations ranging from ∼2 months to up to 4–5 years (23, 30–34). While restrictions regarding prescription and take-home dosing have loosened over time (alongside slow growth in the availability of extended-release formulations), initiation of MOUD often requires daily to weekly clinic visits and in the case of methadone, can only occur at federally-licensed Opioid Treatment Programs (OTPs) (6, 35–38). For many, medications may insufficiently address deficits in psychosocial functioning, comorbid disorders, and/or health-related quality of life (5, 7, 39, 40) and disengagement from MOUD can result in striking increases in mortality risk (23). Though adjunctive programs like psychotherapy and case management can buttress areas unaddressed by MOUD (41, 42), many patients do not receive these services (43); lack of adjunctive service use appears partially due to the challenges of engaging in further resource- and time-intensive care modalities in addition to the existing demands of MOUD (39, 40, 44, 45). Finding alternative methods of supporting patient needs while pursuing MOUD could help further alleviate suffering and clinical burden.

Mobile Health Interventions (MHI) can provide support to this vulnerable patient group while reducing resource burdens on MOUD clinics and providers. MHIs are defined here as smartphone-based software applications (apps) that utilize graphical user interfaces, text, and multimedia messaging to efficiently implement healthcare interventions, their components, or aspects of their supporting infrastructure (e.g., provider-messaging, app-based presentation of health records). MHIs have seen growing interest as a care medium for substance use and mental health disorders given their perceived potential for facilitating care access/flexibility (46–49) and reductions in healthcare costs (50–52). Such interest coincides with notable growth in smartphone ownership across the U.S., which as of June 2024 included ∼91% of U.S. adults and nearly 80% of older adults (53). Smartphone ownership among hard-to-reach populations like unhoused or formerly unhoused veterans and people with substance use disorders ranges from 57%–94% (54, 55). Other studies examining veterans have found mobile device ownership rates between 76% (48) to 91% (56). Though some research indicates interest in mobile apps as a component of care among most veterans (56%–76%) (48), results have also shown such attitudes are not universal and may vary by demographic factors such as rural v. urban living area (57).

MHIs vary widely in focus, structure, and content, but when considering addiction treatment, several specific examples illustrate how they may be designed and used. For example, “RESET-O®” is an FDA-cleared “digital therapeutic” smartphone app that provides short-term (84-day) adjunctive outpatient support to patients receiving MOUD (58). RESET-O aims to bolster retention and includes audio/video lessons drawn from Cognitive Behavioral Therapy for Substance Use Disorders (CBT-SUD) and Contingency Management that are progressively available as patients move through the app. This more structured approach is intended for use in conjunction with formal treatment. Patients using RESET-O have the opportunity to earn rewards (e.g., merit badges, gift cards) for demonstrating treatment engagement and logging of negative drug screens (51). The app “A-CHESS” (Addiction—Comprehensive Health Enhancement Support System) provides a less-structured approach to mobile care offering with “a suite of services that promote positive behavior change and provide 24/7 support.” (59) A-CHESS was developed for patients exiting treatment for alcohol use disorder, though adaptations of this app have been applied to the care of other substance use disorders (60). This app has been used in the context of structured treatment and in conjunction with providers, but also includes a variety of static (e.g., audio-guided relaxation) and interactive features (e.g., GPS alerts if a patient approaches a location they label as “high risk” for substance use) accessible outside formal treatment sessions (61).

Despite the growth in MHI availability, little work has targeted MHI use among veterans with OUD. MHIs could serve as an especially helpful tool for veterans with OUD, providing adjunctive support for many common co-occurring conditions (e.g., pain, PTSD) (5). Additionally, given that the VHA provides treatment to nearly 60,000 veterans with OUD (45, 62) and represents one of the largest integrated healthcare systems in the country, there is also significant potential to scale a low-intensity intervention for wide-spread impact. While a potentially impactful opportunity, successful development of MHIs requires thoughtful consideration during the pre-implementation phase of planning, including careful conceptualization of intended user needs alongside intended-user involvement, to reduce risks of creating unacceptable, inefficacious products that can further exacerbate inequities (63–65). For innovations aimed at behavior change, the COM-B framework is particularly helpful to consider during pre-implementation (66). COM-B offers a systematic method for linking specific intervention functions to behavior change techniques, helping ensure that innovations are tailored to address the actual barriers and facilitators identified within the target population (67). This approach is especially beneficial for developing innovations for new populations, such as MHIs among veterans with OUD, as it would allow for identification of behavior change pathways and selection of appropriate MHI strategies and components prior to implementation.

### Current study

OUD remains a significant public health problem. Despite the availability of efficacious MOUD, sustained engagement in these regimens can be especially challenging and MOUD alone may not address co-occurring psychosocial problems and health conditions. Further, MOUD is often not integrated with adjunctive programs (e.g., psychotherapy), which themselves may be challenging to access. MHIs (i.e., smartphone-based software applications or “apps”) could offer accessible support while reducing clinic resource burdens. However, little work to date has explored how MHIs might be used to support OUD care (particularly among veterans). To address this gap, the current study conducted preliminary work exploring MHI attitudes and design perspectives among veterans receiving MOUD.

## Method

### Study design

Individual semi-structured interviews were conducted within the VA Connecticut Healthcare System (VACHS) between March 2023 and August 2024 in the context of a larger investigation into VHA patient/provider perspectives towards MOUD and the use of digital health tools (HSR&D Virtual Care CORE Project#: COR-20-199-13). Methods for this study have previously been described in the initial paper from this project (40) but are briefly reproduced below. This study and its procedures were approved by the VACHS IRB (protocol#: 1660970).

### Study team

At the time of data collection, our study team consisted of 2 clinical psychology postdoctoral fellows (authors: NRW and CWM), 3 licensed clinical psychologists (authors: EDR, RRM, and GAP), and 1 addiction psychiatrist (author: MS). Study authors worked within VHA hospitals at addiction-focused mental health clinics (*N*RW, CWM, and RRM), pain and general mental health clinics (GAP and EDR), or health services research centers (MS).

### Study setting

The VACHS is a large, integrated healthcare system across the state of Connecticut, comprised of main hospital campuses in West Haven and Newington, as well as several Community-Based Outpatient Clinics (CBOCS) and a medical facility annex. VACHS MOUD is typically given as outpatient prescriptions of oral buprenorphine via weekly or monthly visits. Methadone dosing occurs at the West Haven hospital Opioid Treatment Program (OTP). VACHS MOUD programs generally include a mix of treatment goals targeting MOUD compliance, harm reduction and/or twelve-step approaches, and adjunctive services (e.g., trauma or pain care).

### Sampling & recruitment

Based on prior methodological and qualitative research, our team estimated a required *N* ≥ 20 (68–71). Study recruitment notices were made via announcements at VACHS addiction clinic team meetings, fliers posted throughout hospital common areas, and word-of-mouth. Eligible veterans were VACHS patients actively prescribed buprenorphine or methadone for OUD. According to participant preference, veterans could be interviewed in-person (*n* = 8), virtual teleconference (*n* = 5), or telephone (*n* = 8); for remote interviews, participants were asked to ensure they were in a private, quiet setting. Recruitment occurred until the study team collectively agreed that data saturation and our minimum sampling goal was reached.

### Participants

A total of 25 veterans were approached, all of whom expressed interest and were determined as eligible to participate. Of these, 4 expressed interest but did not attend study visits or respond to follow-up efforts, resulting in *N* = 21 veterans ultimately participating. No participants discontinued interviews after starting. Twenty of the participants in our sample received MOUD through VACHS, while one received MOUD through a community setting and only used VACHS for other healthcare services. Demographic data can be found in Table 1. Interview times ranged from 26 to 80 min (*M* = 44, *SD* = 13).

**Table 1:** Participant demographics (*N* = 21).

Demographic characteristic	*n* (%)			
SAB				
Female	2 (9.5%)			
Male	19 (90.5%)			
Gender Identity				
Female	2 (9.5%)			
Male	19 (90.5%)			
Race				
Black or African American	3 (14.3%)			
White	17 (81.0%)			
Prefer not to answer	1 (4.8%)			
Ethnicity				
Hispanic/Latino/Latina/Latinx	2 (9.5%)			
Non-Hispanic/Latino/Latina/Latinx	18 (85.7%)			
Prefer not to answer	1 (4.8%)			
MOUD Type				
Buprenorphine	11 (52.4%)			
Methadone	10 (47.6%)			

	*M* (*SD*)	Median (IQR)	Min	Max
Age	59.95 (10.50)	62.00 (11.00)	39.00	73.00
Years in Current MOUD Episode^a^	6.43 (5.20)	5.00 (5.00)	0.50	20.00

SAB, sex-assigned-at-birth.

a Current MOUD episode refers to self-reported years from most recent induction to the date participant was interviewed.

### Conceptual framework

Qualitative interviews were grounded in the COM-B model of behavior (66). The COM-B model describes behavior as derived from an individual's Capability (psychological and physical capacity for behavior), Opportunity (physical or social factors outside the individual which make a given behavior possible), and Motivation (including habitual or automatic processes energizing behavior, as well as reflective or analytical decision-making). “MOUD engagement” was broadly the “behavior” considered here, conceptualized as the collection of actions required to comply with and immerse oneself within treatment regimens (e.g., appointment attendance, medication compliance, participation in recommended adjunctive intervention).

### Data collection

We used Rapid Analytic Procedures (RAP) (72, 73) and saliency analysis (74), through semi-structured individual interviews as part of a team-based approach. Interviews were conducted by NRW and CWM (postdoctoral clinical psychology fellows at the time of data collection), who alternated roles between interviewer and note-taker. Data collection and analysis were supervised by GAP, a VACHS research psychologist and qualitative methods expert. Interviews were audio-recorded using Microsoft Teams and transcribed verbatim by the VA Centralized Transcription Services Program.

Prior to data collection, and in accordance with the needs of the parent study and time permitted for each interview, a brief semi-structured interview guide was developed and reviewed by the research team to ensure questions examined critical content areas. This guide used open-ended questions to obtain a broad examination of veterans’ general attitudes and design perspectives towards MHIs in MOUD. NRW and CWM convened following initial interviews (3rd interview) (*n* = 3) to discuss the interview guide's effectiveness, which ultimately resulted in minimal changes (i.e., re-ordering questions) and the revised version was then used in subsequent interviews (Supplementary File S1). Additional study team meetings did not indicate a need for further interview guide changes.

Interviews began with introductions, discussion of interviewer/note-taker qualifications and professional roles, and consenting. Time was allocated for participants to ask questions prior to starting interviews. Once interviews were completed, recording equipment was turned off and participants completed a brief self-report demographics questionnaire. Debriefing between interview and notetaker (NRW and CWM) typically occurred immediately following interview completion (but always within 48 h).

### Data analysis

First, NRW and CWM reviewed a sample of interview field notes to identify initial codes. NRW, CWM, and GAP assessed this initial code-set, refining definitions and organizing them within interview domains to create an interview summary template. NRW and CWM then independently applied this template to the remaining field notes. NRW, CWM, and GAP then re-convened to review resultant codes, including assessing for recurrence and importance (74). Few changes occurred during review and data was then summarized into the themes presented below. Audio transcriptions were referenced while summarizing to verify quote accuracy and concordance with data. Reporting of this research corresponds with the COREQ checklist (75) (see Supplementary File S2).

### Data credibility

We employed several design and procedural approaches to enhance data trustworthiness. To help ensure coding reliability, appropriate determination of data saturation, and attendance to researcher reflexivity, we engaged in the following: (a) the research team collaboratively developed semi-structured interview guides and collectively reviewed them to ensure that questions addressed key content areas prior to interviews; (b) following interviews, two interviewers (NRW and CWM) conducted calibration sessions to evaluate how the interview guide was functioning and discuss any necessary refinements; (c) we alternated the two interviewers in the “interviewer” vs. “note-taker” roles to allow for multiple perspectives and interpretations of data; (d) we included a post-interview debriefing (typically immediately following but always within 48 h of interviews) to provide opportunities for reflection on initial observations and attention to the frequency and importance of data from interviews; (e) we held regular (approximately monthly) research team meetings throughout study preparation, data collection, and data analysis, in which transcript summaries, code recurrence, and code importance were discussed; (f) reviewed audio recordings and transcripts during data analysis to verify pull quote accuracy, alignment with codes, and to ensure interviewer interpretations reflected quote contexts; and (f) we presented preliminary findings (mid-way through and at the conclusion of data collection) at national conferences and VA provider forums where we solicited expert input on study methodology, finding plausibility, and implications.

Data triangulation was achieved through applying a broad and flexible recruitment approach supporting engagement from veterans on MOUD throughout the VACHS hospital system, on both major types of MOUD, and across stages of their recovery. Specifically, this involved (a) recruiting across a variety of MOUD clinics representing different levels of clinical severity, need, and contact (e.g., outpatient buprenorphine clinics, Opioid Treatment Programs, Intensive Outpatient Programs, residential treatment facilities, pain clinics); (b) recruiting across multiple VACHS hospital campuses and clinics, as well as in non-addiction settings (e.g., pain clinics) and common areas (e.g., cafeteria); (c) including participants regardless of MOUD type/formulation (e.g., methadone and buprenorphine, immediate and extended-release), and regardless of their duration in MOUD, and (d) allowing participants to complete the interview through a variety of modalities (i.e., in-person, telephone, or via virtual teleconference).

## Results

### Themes

We identified three overarching themes related to veteran attitudes towards MHIs supporting MOUD care. Additionally, saliency analysis suggested one veteran's opposition to MHI's was important for fully conceptualizing the spectrum of attitudes in the general veteran population. We also identified several themes on veteran perspectives towards MHI design (i.e., *how* MHIs supporting MOUD should function and *what* they should aim to do), which are mapped to the COM-B model of behavior. Participants are distinguished by unique ID numbers (listed following pull quotes), along with the MOUD type a given participant was taking at the time of the interview. Themes are bolded, subthemes are *bolded and italicized,* and *non-thematic codes are bolded, italicized, and underlined.*

### Attitudes towards MHIs supporting MOUD

**Figure 1:**
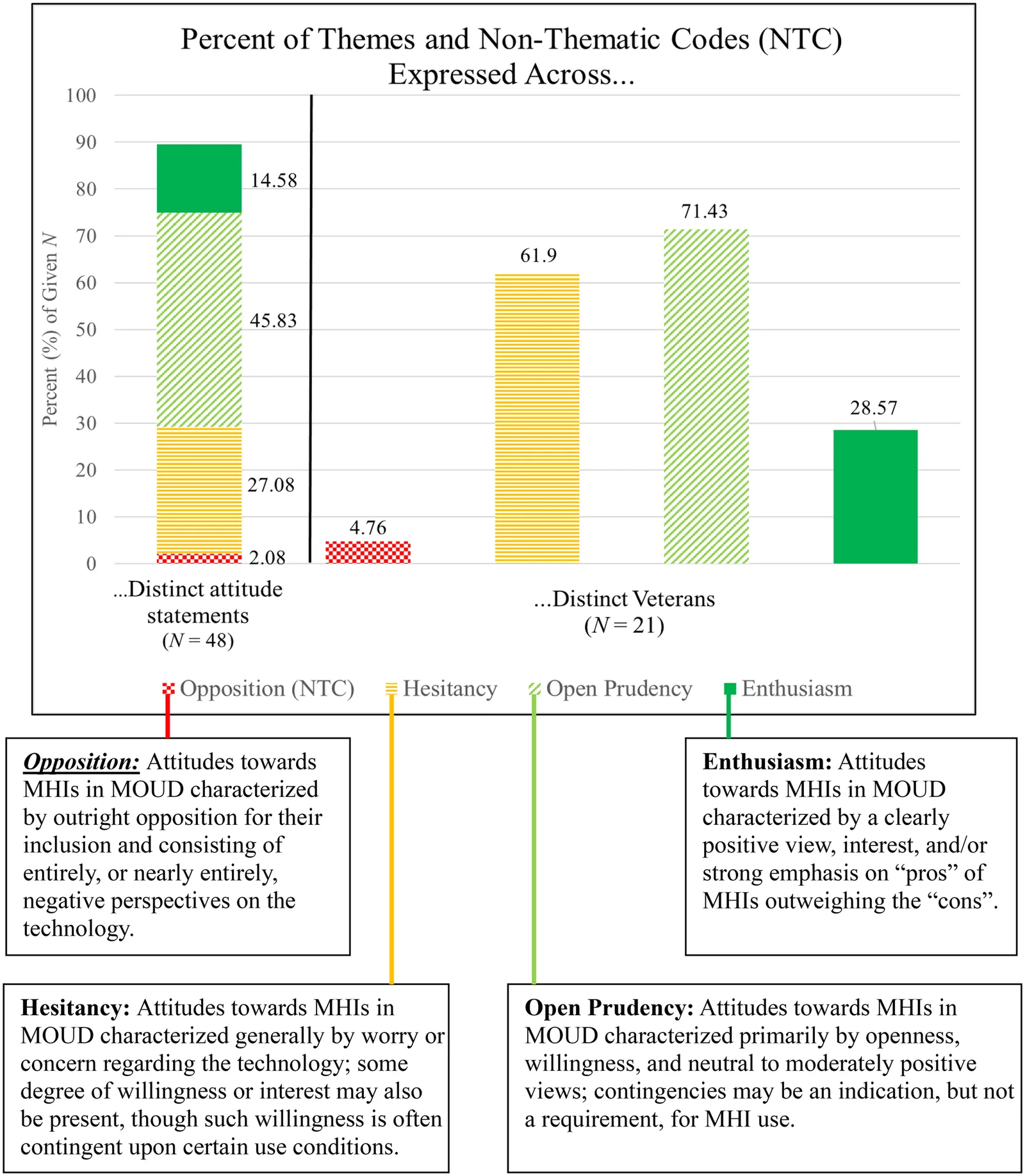
Spectrum of themes and non-thematic codes expressed across total unique attitude statements (*N* = 48) and veterans (*N* = 21) towards Mobile health interventions (MHIs) to support medication treatment for opioid Use disorder (MOUD). As many veterans simultaneously expressed positively- and negatively-valanced attitudes towards MHIs in MOUD (e.g., a veteran noting they like the idea of a more flexible treatment medium while also citing concerns about privacy and information security), some Veterans were classified for more than one Theme. To acknowledge this fact while preserving unique data elements, we present both unique Veterans (*N* = 21) on the righthand side of the figure and identified distinct attitude statements during interviews (*N* = 48), presented on the lefthand side.

Many veterans simultaneously expressed positively- and negatively-valanced attitudes towards MHIs in MOUD (e.g., a veteran noting they like the idea of a more flexible treatment medium while also citing concerns about privacy and information security). To acknowledge this fact while preserving unique data elements, we identified distinct attitude statements during interviews (*N* = 48), which were then examined with respect to content and valance. The spectrum of veterans’ attitudes is illustrated in Figure 1 below and subsequently discussed, beginning with the most negatively valanced and moving to the most positively valanced.

One veteran expressed homogenous, outright Opposition to MHIs in MOUD. Though infrequent, the homogenous and strong negatively-valenced attitudes expressed by this veteran indicated concerns about the potential value of MHIs and data security that were described to a lesser degree by other veterans in the sample (discussed below).

“What can a smartphone do for me? I’m in addiction. And to put my business on a phone so whoever hacks it can see what's goin’ on in my life… why would I wanna do that?” –Veteran 19, methadone

More commonly, however, negative attitudes towards MHIs in MOUD appeared to reflect hesitancy more so than direct opposition. The valence of these perspectives ranged from mostly negative to neutral and were characterized by worry or concern, though some degree of willingness or interest (frequently contingent upon certain use conditions) was often simultaneously present. Concerns about MHI *Inaccessibility & Pragmatics* were often cited, in which participants noted certain care elements could not be done via digital mediums (e.g., providing toxicology screens) and felt other potential benefits could be negated if the needed technology was not inherently provided.

“… if they [VHA] made tablets and phones readily available… that would help a lot.” –Veteran 7, buprenorphine

“What if someone doesn’t have a phone? And [can’t pay] no bill. If you’re going to use that and it depends on you paying a bill, people in recovery don’t always have money.” –Veteran 10, buprenorphine

“How many addicts have phones?” –Veteran 13, buprenorphine

Relatedly, several veterans described *Technological Inefficacy*, noting concerns that MHIs might only be accessible to technologically-savvy patients and could be frustrating to those less familiar or less adept. Nonetheless, participants indicating this theme often reported willingness to use MHIs provided technical support was also available.

“Change is never easy for me… if I was assured that someone was there to show me how to use it… otherwise, I would get frustrated… and feel like I’m being left behind because I’m older.” –Veteran 10, buprenorphine

Additionally, many veterans expressed concerns about *Information Security*, emphasizing that clarity in understanding what data was collected and how it was protected as critical to their willingness to use MHIs.

“I’d be for it, as long as it was anonymous.” –Veteran 6, methadone

Finally, several participants reported skepticism that MHIs could overcome *Intrinsic Motivational Barriers* present when seeking addiction care. While many reported interest and willingness to use MHIs as part of their MOUD, participants here simultaneously described that other patients “have to want it” and felt in the absence of this motivation, MHIs were not likely to be used or if they were used, may not be efficacious.

“If you don’t have it in your mind [to commit to recovery]… no matter what technology you have it’s not gonna work.” –Veteran 4, buprenorphine

Most frequently, veterans reported Open Prudency, describing neutral to moderately positive attitudes towards MHIs in MOUD characterized by an openness or willingness to engage with such technology; while contingencies for MHI use might be indicated, such contingencies were not described as preclusions. *Open-Mindedness* was a predominant subtheme, with participants regularly expressing desires to “try out” and explore MHIs as a means of supplementing MOUD while simultaneously acknowledging pros and cons.

“I think [MHIs in MOUD] would be a good step… I would see what’s going on with it.” –Veteran 18, methadone

Many saw MHIs as a means for increasing care *Flexibility & Individualization*, noting they could increase care choice and reduce certain access barriers (e.g., participating in a virtual therapy group, receiving treatment documents electronically, digital messaging with providers). Other considerations raised included *Preferences for In-Person Care*, in which interest in learning about or trying MHIs was viewed as far more acceptable if access to in-person modalities for care was preserved. Explanations for this preference included perspectives that in-person connection facilitated better outcomes and feelings of connection to current care providers.

“I’m on board for it [using MHIs in MOUD] because that’s the way our world is going. I always think in-person immediate connection is going to be the best tool… but for those limited or unable to get where they need to… I think [apps] are beneficial to bridging gaps.” –Veteran 14, buprenorphine

A small but notable proportion of veterans reported Enthusiasm for MHIs in MOUD, expressing clearly positive attitudes. Such participants stressed the potential for MHIs to *Enhance Care Engagement* through limiting requirements for in-person visits and reducing constraints around appointment scheduling. Most participants expressing enthusiasm for MHIs referenced their own *Technological Fluency* as an important factor. These veterans described how their existing familiarity and competency with digital technologies, and the general perceived technical fluency of others, would result in easy incorporation of MHIs within their care.

“I think it’s [using MHIs in MOUD] fantastic. I think it’s absolutely 100% right on the money.” –Veteran 8, methadone

“… I think it’s [MHIs] a good thing. I think technology has come a long way and I think counselors are able to be more supportive. More in each person’s treatment.” –Veteran 24, methadone

“I think everybody’s tech savvy enough to get a phone and log into a meeting [within an MHI]… for the most part, society can do that.” –Veteran 7, buprenorphine

### Perspectives on MHI design and function

**Figure 2:**
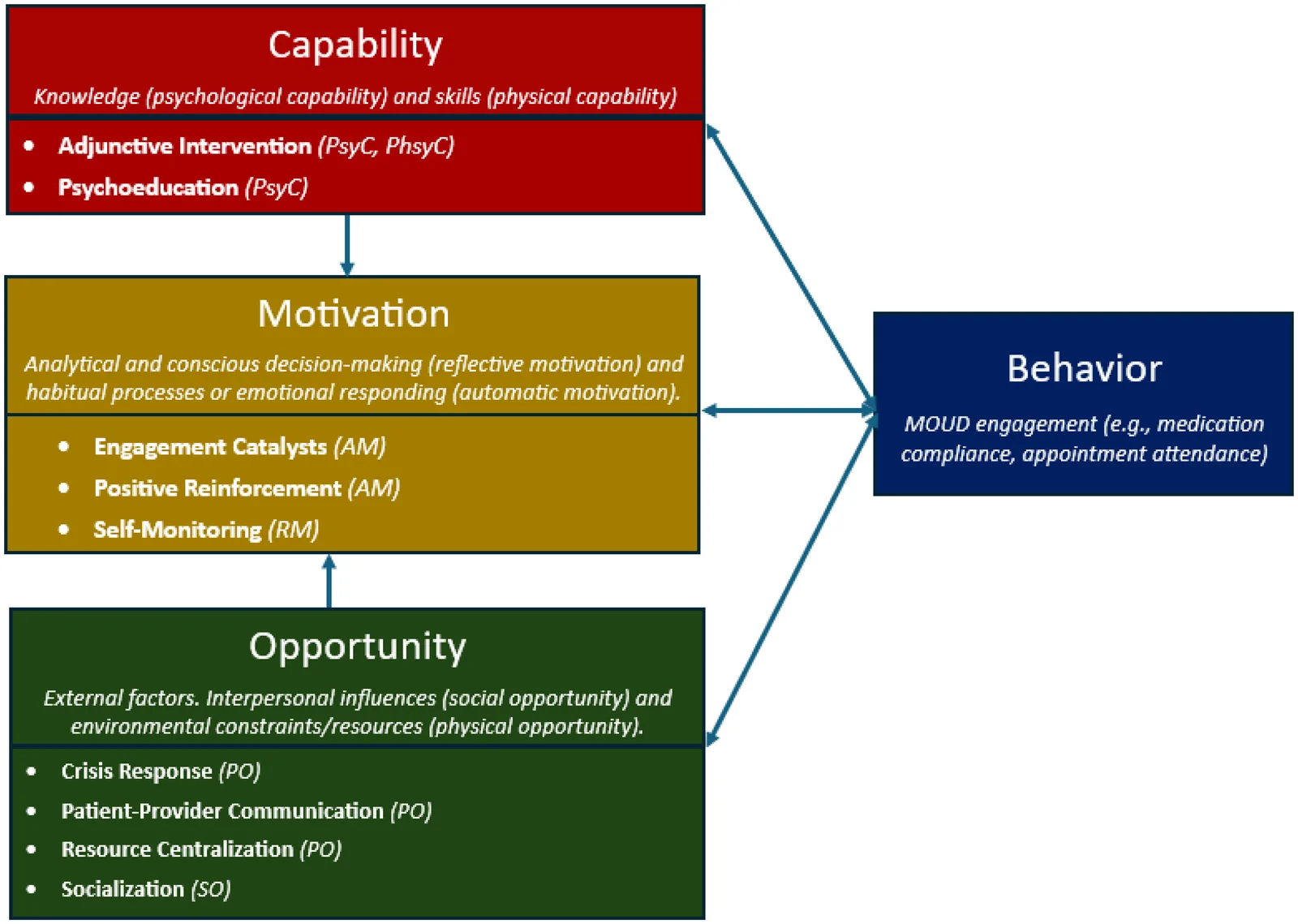
Mapping of MHI design themes to michie et al.’s (66) “capability, opportunity, motivation model of behavior” (COM-B). PsyC, psychological capability; PhyC, physical capability; RM, reflective motivation; AM, automatic motivation; SO, social opportunity; PO, physical opportunity.

Questions regarding design of MHIs in MOUD focused on what veterans thought this technology should do and how they thought it should serve these functions within their care. After identification, themes were then mapped to the domains/subdomains of COM-B model of behavior (66). Figure 2 below illustrates the mapping of themes to the COM-B model and is followed by description of these themes.

### Capability

We first considered themes indicating aspects of MHIs which would facilitate physical capability (e.g., taking MOUD medication as scheduled, practicing addiction-related coping skills) and psychological capability (e.g., understanding of MOUD regimens, knowledge of addiction's etiology and sequalae) to support MOUD engagement. These included using MHIs to provide Adjunctive Psychosocial Interventions (or their components) and Psychoeducation in support of improving one's own ability to manage aspects of OUD and comorbid conditions. Examples from veterans included access to digital copies of “The Big Book” from 12-step approaches as well as information on addiction etiology and symptoms and the broad structure/process of MOUD care:

“Technology is great, isn’t it?. I can show you some of the [apps] I use… this is the Big Book… if I need it, I’ve got my motivational speakers on here… any time I want to share something, I can go here and search for it and share it.” –Veteran 12, buprenorphine

“Especially… if they don’t know if they have a problem or not… that [an MHI] could lead ‘em to [ask] ‘Is this happening?’, ‘What do you look for?’, the warning signs, the helpful signs, and different things like that.”–Veteran 3, buprenorphine

### Motivation

When mapping themes to the COM-B motivation domain, we considered aspects of MHIs which would energize treatment-related behaviors, whether through intentional, analytic, or conscious processes (i.e., reflective motivation) or habitual and emotional responding (i.e., automatic motivation). These included using MHIs as Engagement Catalysts by targeting their use during periods such as early care or during psychosocial stressors (e.g., MOUD induction, transitions between medications, following the loss of a loved one or job); in doing so, participants indicated this would couple an intervention with a relatively lower-threshold for use when need for support might be higher. Further, participants stressed the importance of integrating Positive Reinforcement within MHIs to encourage treatment-related behavior (e.g., inserting motivational quotes, gamifying elements of the MHI to give rewards for treatment engagement or progress) and providing a means for Self-Monitoring impairing symptoms or successes within care (e.g., in-app trackers for craving symptoms or continuous days of sobriety from illicit substances).

[When asked what an MHI in MOUD might best be able to help with:]

“Let’s say when they’re early in recovery and still getting urges. A stressful situation where they can reach out and right away get into the app and get some pearls of wisdom.” –Veteran 23, buprenorphine

“I’d make sure there was a reward system, not a punitive system.”–Veteran 17, methadone

“… they could set it up where you could keep track of your clean dates.” –Veteran 24, methadone

### Opportunity

Finally, we examined how participants saw MHIs being used to influence social and cultural factors relevant to treatment (i.e., social opportunities) and environmental restraints/resources (i.e., physical opportunities). Many participants indicated interest in Resource Centralization as an invaluable function of MHIs within MOUD. Here, veterans described how the wider-ranging consequences of addiction, including housing and food insecurity, employment instability, or ongoing legal cases, could substantially interfere with treatment and recovery. They also shared how finding support for such problems often extended beyond what was available within MOUD clinics. Thus, participants indicated MHIs that could collate information for local resources in these areas could be especially helpful.

“… when you start in recovery, there’s a lot of things you need. You need financial help. You need to have a job… You gotta be able to do it yourself and… you can use the smartphone for that.” –Veteran 3, buprenorphine

“… you need… practical things that people need help with… do you need food? Okay, this is where you go at this day and this time, walk in there, show them this [MHI]… just make it very simple for ‘em cause in the beginning I think people need that very much.” –Veteran 8, methadone

“And a resource tab [in an MHI]… So you clicked on family resource, and then in there, it would give you… providers and family support specialists. Upcoming events in your area.” –Veteran 14, buprenorphine

Participants also expressed interest in MHIs that facilitated Patient-Provider Communication (e.g., instant-messaging platforms) to alleviate confusion and challenges in identifying and initiating contact with providers, as well as directly communicating with providers outside of scheduled appointments.

“When I get on that [MHI], I wanna hear, ‘you can come here tomorrow and I’ll help you’… or ‘You want help? Here’s the phone number’… The goal is to get the person connected to someone that cares about them as quickly as possible.” –Veteran 24, methadone

Crisis Response tools were also routinely described as a desirable function of MHIs for MOUD. Participant responses suggested such tools would be somewhat distinct from emergency services such as 9-1-1 or suicide hotlines where emergency medical services for life-threatening situations might be sought, and instead typically described as providing an option for rapid contact with individuals familiar with addiction offering validating treatment support.

“… if you could use your smartphone… [to] call your go-to person if you’re feelin’ a relapse is on… [or] you don’t always have to talk to someone, but even just a message to a computer to say ‘I’m still hangin’ in here’.”–Veteran 21, methadone

“People who don't have like families or support system like I had, it may have been nice to be able to have that phone to just hit a button and call somebody who cares. You know? I didn't have that.”–Veteran 24, methadone

Lastly, participants often stressed the importance of using MHIs to provide opportunities for Socialization with others receiving MOUD. This could include text-based communication through app-based message boards, forums, or other chat-based tools. Veterans here underscored the value of “lived experience” in addiction and how connection with such peers increased the perceived legitimacy of the app and their sustained interest in treatment.

[When asked what they would prefer to have available in the app:]

“It helps to have people who have been through it before, it really does. The honesty that you get from them is unbelievable… [they] trashed their own life once, maybe they'll help me stop trashing my life.” –Veteran 2, buprenorphine

## Discussion

This qualitative and hypothesis-generating study examined the attitudes and design perspectives on MHIs in MOUD among veterans receiving MOUD this treatment. These data serve as an important initial step in exploring optimal approaches for MHI design and later implementation. Results suggest that veterans receiving MOUD were broadly interested in the potential inclusion of MHIs within their care, while underscoring the necessity of attending to intended users’ concerns throughout development. Findings also indicated that optimally designed MHIs for veterans with OUD would integrate support for psychosocial functioning and engagement in existing care structures, alongside MHI-specific features for symptom management.

Veteran attitudes towards MHIs in MOUD generally reflected openness and curiosity, with such tools being seen as an opportunity to increase the flexibility and individualization of their existing care. Notably, while digital tools to provide intervention or intervention-related content were described (e.g., MHI-delivered psychosocial modules for coping with cravings, psychoeducational pdfs on MOUD medications), most veterans described MHIs as tools to integrate within and support existing care structures (e.g., rapid connection during crises to local providers familiar with addiction; collation of local resources for housing, occupational, or legal supports; easier instant-messaging with their current treatment team). Previous studies have similarly found most veterans appear open to the use of MHIs and digital technologies within their care (48, 54); however, most MHIs developed for individuals with substance use disorders to-date have prioritized symptom reduction and management over psychosocial functioning and adjunctive resource connection [e.g., RESET-O (58) utilizes CBT-SUD and contingency management, A-CHESS (59) provides a suite of coping related tools such as audio-guided relaxation and GPS alerts when near “high risk” areas for substance use]. While features connecting patients to treatment communities (e.g., digital forums) have occasionally been included in MHIs for addiction populations (76), simultaneous availability of functional- and resource-support tools is uncommon, and it is even rarer that an MHI with such features is well-integrated within existing care systems.

Our findings also highlight several important potential user concerns which could significantly inhibit MHI adoption, use, and/or effectiveness if ignored. For example, concerns about privacy, data security, and technical support were prevalent, despite our sample representing participants receiving care within an integrated healthcare system that has dedicated technical support offices [e.g., the VA's Office of Connected Care (77)] and which prioritizes dissemination of digital privacy practices (e.g., (78)). Such concerns may reflect broader population experiences with digital technology and mobile applications in particular; most commonly-used apps do not have privacy policies and those that do tend to present lengthy articles written at high grade levels that do not reference the apps to which they are attached (79) [though notably, research centers within VHA are striving to counter such problems (80)]. Thus, while there appears to be a willingness and interest in using MHIs, development should be grounded in clear, simple explanations of the app's purpose, function, and use structure. Similarly, adoption and uptake would likely be improved if such information was presented in advance to intended users, especially by providers, and if ongoing technical support were available (81).

Additionally, veterans in our sample frequently referenced the importance of user-friendly and aesthetically pleasing presentations that incorporated positive reinforcement throughout and were targeted during periods where desire for care may naturally be greatest (e.g., early in MOUD initiation, during psychosocial stressors). The former strategy is widespread throughout contemporary mobile app design and includes simple strategies like “streak” notifications or motivational messages (82), as well as more advanced and resource-intensive approaches such as the delivery of retail gift cards based on app progression (58). Despite its ubiquity, the application of reinforcement within MHIs is inconsistent and findings from several studies indicate it may not improve treatment outcomes (33, 82–84). Given the potential complexity of reinforcement structures (e.g., token-economy based reward systems, which often require developer-determined values that are progressively unlocked through engagement with features across different app sections), unscrupulous attention to this area risks over-complicating MHI design, substantially escalating development costs, and reducing feasibility. More experimental and prospective research is needed to delineate how to best implement app-based reinforcement within MHIs.

Collectively, developing an MHI that includes features for symptom management and functional support, which also facilitates engagement in existing MOUD care, is a challenging task. Environments which may be best suited for this intervention modality ideally would have mechanisms to fund high early development costs, structures for app hosting, dissemination, and for receiving user feedback, and the availability of integrated interdisciplinary care. To provide an illustrative example, consider the VHA, which is a large integrated healthcare system that has funded system-wide MHI development and distribution infrastructure [e.g., the VA's Office of Connected Care (77) and its “VA Mobile” (85) department, the Center for Mobile Applications Research Resources & Services (86)]. Within this setting, a VHA-based MHI for veterans in MOUD might include psychoeducational materials (e.g., on service connection for functional support) and symptom management tools (e.g., craving trackers) that can be used in conjunction with traditional MOUD care. Integration of this app with electronic health records and inter-departmental systems could reinforce treatment engagement (e.g., in the form of earning in-app coupons to VHA canteen stores where basic necessities and recreational goods might be purchased). Such integration would also allow for personalization of app features and reinforcement scaling based on personalized risk markers (e.g., homelessness, recent medical diagnoses, time since care initiation). Notably, however, this is an illustrative and idealized example. Future work examining how different healthcare system characteristics impact MHI dissemination, implementation, adoption, and effectiveness is needed.

Comprehensively addressing *all* of these elements in a user-friendly fashion within a single MHI may not be necessary and in many contexts is likely infeasible. Instead, more focused apps might demonstrate clearer application/benefit to patients and providers, while allowing for less cumbersome development, dissemination, and implementation. Design of such apps should ideally reflect the most prominent needs of a given patient population. For example, participant responses here ultimately reflected elements of the COM-B model of behavior, underscoring desires for MHIs which facilitate their capability to engage within MOUD, reduce treatment barriers, and catalyze motivation for care. Thus, it may be more helpful and realistic to use goals such as these to frame future MHI design. Critically, as this work was conducted within a single VHA hospital, use of these goals should be integrated alongside considerations of local clinic and healthcare system environments and involve input from local patient populations (63).

### Limitations

Our study had several limitations important to note. First, our sample represents a homogeneous group of predominantly White (Non-Hispanic/Latinx), cisgender male participants from VACHS MOUD clinics. Despite some concordance with studies examining mobile app attitudes in larger samples using substances (54) and among veterans (48), other investigations have found starker divides in attitudes which may reflect rural vs. urban differences (57). Thus, results may not generalize to other populations and verification of the findings observed here with larger, more diverse samples (e.g., national attitude surveys) is an important goal for future research. Other researchers have also developed frameworks that future investigators could use to improve MHI access, adoption, and uptake (63). Second, recruitment materials for the study noted that it would involve asking questions about attitudes and perspectives towards MHIs; thus, individuals who were uninterested in MHIs could have self-selected out of participation, while conversely, those more interested may have been more likely to participate. Though we attempted to reduce the potential impact of such bias by incorporating less common, but nonetheless important, data via saliency analysis (74), future studies might consider targeted sampling strategies and recruitment materials that enlist participation from individuals who are less interested in MHIs. Third, technological fluency was not formally assessed as part of our interviews and could have influenced study findings, as suggested by the “Enthusiasm” attitude theme. Limited digital literacy may reduce interest and/or increase anxiety around using MHIs. As a result, higher rates of digital *il*literacy among intended user groups could have significant implications for if/how an MHI (or any digital health intervention) is implemented (e.g., the level of provider involvement needed, the degree/availability of technical support provided). While our sample better represents the attitudes clinics might encounter implementing MHIs in the “real-world”, where treatment is not restricted based on technology ownership/fluency, systematic examination of how these variables impact MHI attitudes and use remains a critical target for future research. Fourth, this study did not include MOUD providers or hospital administration. The absence of such data potentially limits data triangulation and assessment of these groups would be essential to ensure MHI development adheres to healthcare system constraints and aligns with the treatment approaches/environments in which it would be deployed. Fifth, while the rapid qualitative methods allowed efficient completion within pragmatic constraints, this approach could reduce analytic depth. Follow-up work employing alternative qualitative approaches [e.g., inductive approaches (87)] would be valuable in ensuring the full context of digital intervention use within MOUD treatment is captured. Finally, this research did not examine an actual MHI; while our data provide critical grounding, future MHI development should include completion of acceptability, feasibility, and efficacy testing.

## Conclusion

Results from this qualitative and hypothesis-generating investigation of U.S. military veterans provide important preliminary data to guide future development of MHIs for supporting MOUD engagement. Veterans receiving MOUD generally appeared open to and interested in using MHIs as part of their care regimens, and MHIs were broadly described as potential means to enhance treatment accessibility, convenience, and individualization. Nonetheless, such openness appeared significantly bolstered by, and sometimes contingent upon, MHIs whose purpose, function, and data security practices were clearly and simply explained, with interactive technical support, and when integrated as an adjunctive care option (vs. as a requirement or replacement for traditional care modalities). Desired MHI functionality was multi-faceted and included tools for promoting self-management capabilities within MOUD (e.g., psychoeducation, adjunctive intervention), enhancing opportunities for engagement (e.g., direct provider messaging, social forums for treatment peers), and as catalysts for treatment motivation (e.g., targeting implementation during periods of high urgency for treatment such as MOUD induction). While there is potential for the use of MHIs as a patient-centered and accessible MOUD care support, additional work is needed to further understand their clinical impact and what healthcare system characteristics best facilitate their development, distribution, and revision.

## Data Availability

The datasets presented in this article are not readily available because this would risk violation of participant privacy and confidentiality. Requests to access the datasets should be directed to Noah.Wolkowicz@va.gov.
