# Supplementary material for: Engaging veterans in the development of mobile health interventions to support medication treatment for opioid use disorder
Source: Front Digit Health. 2026 Sep 4;8:1805086. doi: 10.3389/fdgth.2026.1805086 (PMC13585756; doi:10.3389/fdgth.2026.1805086)
Supplement: Supplementary file 1 [file Datasheet1.pdf]

Supplementary Appendix S1. Semi-structured qualitative interview guide.

NOTE: This study occurred in the context of a larger qualitative investigation (Wolkowicz et al., under review) into veteran-perceived barriers/facilitators to Medication treatment for Opioid Use Disorder (MOUD). We present the full interview guide for transparency. Interview questions pertaining to MHIs in MOUD are at the end of the interview guide and unshaded; the remaining interview questions pertaining to MOUD barriers/facilitators are shaded gray.

Qualitative Interview Guides – MOUD Patients

Estimated Time: 60-90min

[if time, get demographics answered, at least MOUD Type to help guide interview]

Suggested Orientation Script: *"Thank you for taking the time to talk with us today! My name is ______ (interviewer) and this is _______ (observer). _______ (observer) will be observing our interview today and taking notes. I'll be asking some questions today about Opioid Use Disorder ("OUD") treatment. When I say "OUD treatment", I'm talking about medications prescribed to help people manage OUD like buprenorphine or methadone. Specifically, I'll be asking about your experiences in treatment for OUD and what can make it easy or challenging to stay in treatment. I'll also be asking you questions about using digital health tools, things like smartphones and apps, to improve OUD treatment. The information you provide will help us understand what can make OUD treatment challenging and how digital health tools might be able to help.*

[check if any questions]

There are no 'right' or 'wrong' answers to these questions. Your participation is completely voluntary and you are welcome to skip any questions you don't want to answer. Your name will not be connected to your responses and any information shared with others will only be shared anonymously or in aggregate. I'd also like to remind you that your answers to these questions will not impact your treatment or compensation for participating in this study in any way. As a reminder, you'll be compensated $45 for your participation.

Finally, with your permission, we would like to audio record our conversation so that we can listen back to it in the future. If there is anything you would like us to treat as private, we can stop recording at any time. Do you agree to participate and to be audio recorded?

Do you have any questions before we begin?"

[Start recording] "The interview is now being recorded. The date/time is ____ and this is patient interview #____. If you wouldn't mind repeating, do we have your consent to audio record our conversation?"

[NOTE: Critical questions are highlighted in GREEN. If time is running short, MAKE SURE THESE QUESTIONS ARE ANSWERED.]

[interview questions start on next page]

CONSTRUCTS & QUESTIONS	*NOTES*
**BARRIERS & FACILITATORS TO MOUD RETENTION**	
Sub-domain: [Direct questions about treatment barriers/facilitators]	
What was starting OUD treatment like for you?<br><br>PROBES:<br>• Thinking back on your early care, what made early OUD treatment challenging?<br>• Were there things you were personally dealing with that made starting OUD treatment hard?<br>• What would have made things easier?<br>• What made you want to start OUD treatment? Did that change after you started?	
Since starting out, what have made it challenging to *stay* in OUD treatment?<br><br>PROBES:<br>• Day-to-day, what kind of challenges do you run into with your OUD treatment?<br>• [If primarily pragmatic barriers described so far:] Imagine getting OUD tx was easy (e.g., no transportation problems, it aligned with your schedule) would there still be things that make staying in OUD treatment hard? What kinds of things?<br>• What kinds of things make it *easier* to stay in OUD treatment? What kinds of things have helped you?	
Sometimes people will stop treatment and then start again later. Have you ever started and stopped treatment?<br><br>• [If indicated:] How long have you been in treatment this time around?<br><br>[If yes to the above, ask:] Typically, what has caused you to stop treatment in the past?<br>• Did you talk to anyone before you decided to stop treatment? If so, who and what did they say?<br>• What caused you to go back to treatment?	
Sub-domain: [Experiences with MOUD Meds that Impede/Facilitate Retention]	

What problems has OUD treatment helped with?<br><br>PROBES:<br>• What were things like for you *before* starting OUD treatment?	
What problems *<u>hasn't</u>* OUD treatment helped with?<br><br>PROBES:<br>• What problems do you still deal with, even after taking OUD medication?<br>• What problems would you like to have more help from your OUD providers with?<br>• How do these problems impact your desire to *stay* in OUD treatment?<br>• What kinds of things could you imagine, would make it easier to deal with these problems?	
Sub-domain: [Experiences with Stigma in MOUD that Affect Retention]	
Have you ever experienced stigma related to your OUD treatment (stigma = negative stereotypes or discrimination about people in OUD treatment)?<br><br>[If yes to the above ask:] How did this stigma impact your OUD treatment?<br><br>PROBES:<br>• How did you deal with this stigma?<br>• What would have made it easier for you to deal with this stigma experience?	
Generally, how do you think providers view patients coming in for OUD treatment?	
**SUCCESS IN MOUD**	
When people start out in OUD treatment, what do you think is most important for them to do if they want to stay in treatment?	
How would you define "success" in OUD treatment?<br><br>What do providers need to understand if they want their patients to be successful?	

**FOLLOW-UP**	*NOTES*
Is there anything else about factors that make it hard to stay in OUD treatment I haven't asked about/you'd like to share with me?	

"Thank you for answering those questions! I'd like to shift gears and ask you some questions about using technology to help support people getting OUD care. Specifically, I'm going to ask about using smartphones and apps to support OUD care." (If don't know what smartphone/app is shown on phone or give phone as computer is)

Digital Health Tools = DHTs

CONSTRUCTS & QUESTIONS	*NOTES*
**ATTITUDES TOWARDS MHIs IN MOUD**	
Generally, what do you think about the idea of using smartphones to help people get OUD treatment?<br><br>PROBES<br>• How would you react if a provider asked you to use your smartphone or an app to help support your OUD treatment?<br>• [If concerns for finances and costs indicated as a preclusion:] What if cost was no object or was this technology provided free-of-charge? Would this change what you think about using smartphones/apps in OUD treatment?	
**DESIGN AND USE OF MHIs in MOUD**	
What kinds of problems do you think smartphones or apps could help people in OUD treatment with?<br><br>PROBES:<br>• Imagine you are a CEO at a big tech company. If you were to design a smartphone app for patients in OUD treatment, what kinds of things would you want to make sure it included?<br>• Thinking about your own experience starting and trying to stay in OUD treatment, how could a smartphone app have helped (if at all)?	
What would you want to know before using a treatment app for OUD?<br><br>PROBES	

• What would make you more willing to use a smartphone app to support your OUD treatment?	
**FOLLOW-UP**	*NOTES*
Is there anything else about using smartphones or apps in OUD treatment you want to share with me?	

**GENERAL FOLLOW-UP**	*NOTES*
Is there anything else about anything we've discussed today you want to share with me?	

This concludes the interview. Thank you for talking with us today and sharing your experiences!
